# Recent Research Advances in Mitosis during Mammalian Gametogenesis

**DOI:** 10.3390/cells8060567

**Published:** 2019-06-10

**Authors:** Jia-Hao Wang, Yan Li, Shou-Long Deng, Yi-Xun Liu, Zheng-Xing Lian, Kun Yu

**Affiliations:** 1 Beijing Key Laboratory for Animal Genetic Improvement, National Engineering Laboratory for Animal Breeding, Key Laboratory of Animal Genetics and Breeding of the Ministry of Agriculture, College of Animal Science and Technology, China Agricultural University, Beijing 100193, China; jiahaowang95@163.com (J.-H.W.); scauly@cau.edu.cn (Y.L.); lianzhx@cau.edu.cn (Z.-X.L.); 2 CAS Key Laboratory of Genome Sciences and Information, Beijing Institute of Genomics, Chinese Academy of Sciences, Beijing 100101, China; 3 State Key Laboratory of Stem Cell and Reproductive Biology, Institute of Zoology, Chinese Academy of Sciences, Beijing 100101, China; liuyx@ioz.ac.cn

**Keywords:** mitosis, mammalian gametes, cyclins/Cdk

## Abstract

Mitosis is a highly sophisticated and well-regulated process during the development and differentiation of mammalian gametogenesis. The regulation of mitosis plays an essential role in keeping the formulation in oogenesis and gametogenesis. In the past few years, substantial research progress has been made by showing that cyclins/cyclin-dependent kinase (CDK) have roles in the regulation of meiosis. In addition, more functional signaling molecules have been discovered in mitosis. Growing evidence has also indicated that miRNAs influence cell cycling. In this review, we focus on specific genes, cyclins/Cdk, signaling pathways/molecules, and miRNAs to discuss the latest achievements in understanding their roles in mitosis during gametogenesis. Further elucidation of mitosis during gametogenesis may facilitate delineating all processes of mammalian reproduction and the development of disease treatments.

## 1. Introduction

Gametogenesis is an essential biological process to produce heritable haploid gametes in mammalian gonads, which includes oogenesis and spermatogenesis [1,2,3]. Between eight and nine days post-coitum (dpc), the majority of primordial germ cells (PGCs) are arrested at the G2 phase of the cell cycle [4]. In the germ cell proliferation stage, mouse PGCs enter into the genital ridge by migrating along the endoderm and mesentery of the hindgut, during which PGCs proliferate to form a certain number of germ cells via mitosis [5,6,7]. After arrival at the genital ridge, germ cells not only differentiate into spermatogonia or oocytes to enter gametogenesis but also form the syncytium in which multiple cells share one cytoplasm because of incomplete mitosis [8]. The syncytium contains a mass of cytoplasm that has many nuclei but no internal cell boundaries due to a series of incomplete cell division cycles. Before entering the genital ridge, there is no difference between XX (female) and XY (male) PGCs. However, starting at about 12.5 dpc, female and male PGCs begin to diverge dramatically [9]. In male mice before spermatogenesis, the mitosis of germ cells stops at the G0/G1 phase, which resume division when germ cells become spermatogonia after birth [8]. After mice are born, their germ cells begin to differentiate into spermatogonia via a series of mitotic cell divisions [10]. Conversely, in the female ovary, the mitosis of germ cells continues. Germ cells enter their first meiosis at 13.5 dpc and are then arrested in the diploid phase at 17.5 dpc [11]. During meiosis, homologous chromosomes align and pair with the synaptonemal complex formation to undergo homologous recombination. Then, oocytes and spermatocytes also generate haploid germ cells [12,13].

It is clear that mitosis is an essential process for PGC migration and auxocyte proliferation. Mitosis, a phase of the cell cycle, involves the following processes by which chromosomes migrate to the middle of the cell simultaneously and segregate into two daughter cells equally through the mitotic spindle before cytokinesis [14]. There are four phases in the cell cycle: G1, S, G2, and M. The G1 phase is alternatively called the pre-replicative phase, during which some mRNAs and proteins required for other phases are synthesized [15]. The S phase involves DNA replication, histone synthesis, and nucleosome replication [16,17,18]. In the G2 phase, active cell growth and protein synthesis ensures that cells enter mitosis. The M phase includes both mitosis and cytokinesis to separate the genome and ensures that the two daughter cells inherit an equal and identical complement of chromosomes [14,19]. It is exceedingly complex and highly regulated. Therefore, the M phase is usually divided into five phases: prophase, prometaphase, metaphase, anaphase, and telophase. Prophase, the first phase of mitosis in mammalian cells, includes the condensation of chromosomes, movement of centrosomes, formation of the mitotic spindle, and nucleoli breakdown. Afterwards, the nuclear envelope breaks down, and the chromosomes inside form protein structures named kinetochores during prometaphase [20]. Kinetochores are protein structures that form at the centromere during cell division and attach the chromosomes to the spindle fibers. In metaphase, chromosomes align along the metaphase plate and attach to microtubules anchored to centrosomes which duplicate in the S phase but separate in mitosis [21]. During anaphase, the separated sister chromosomes move from the center of the spindle in the metaphase plate toward opposite poles of the cell (anaphase A), and the mitotic spindle fibers elongate (anaphase B) [22]. Finally, as chromosomes reach the cell poles, a nuclear envelope is reassembled around each set of chromatids, nucleoli reappear, and chromosomes begin to decondense back into the expanded chromatin that is present during interphase.

## 2. Mitosis of Male Gametogenesis

**Figure 1:**
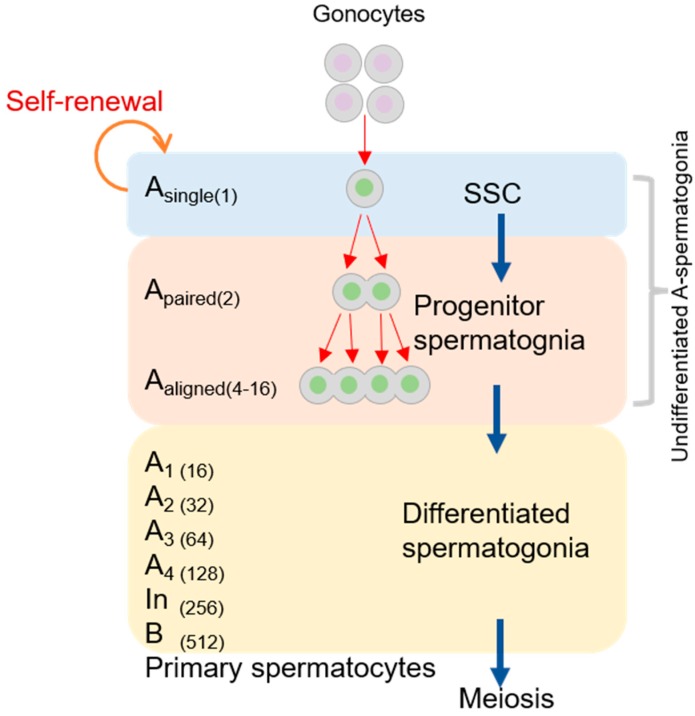
Characteristics of mammalian spermatogonial stem cell (SSC) development. Gray areas correspond to the cytoplasm, dark gray areas correspond to the cytomembrane, lavender and green areas correspond to the nucleus.

**Figure 2:**
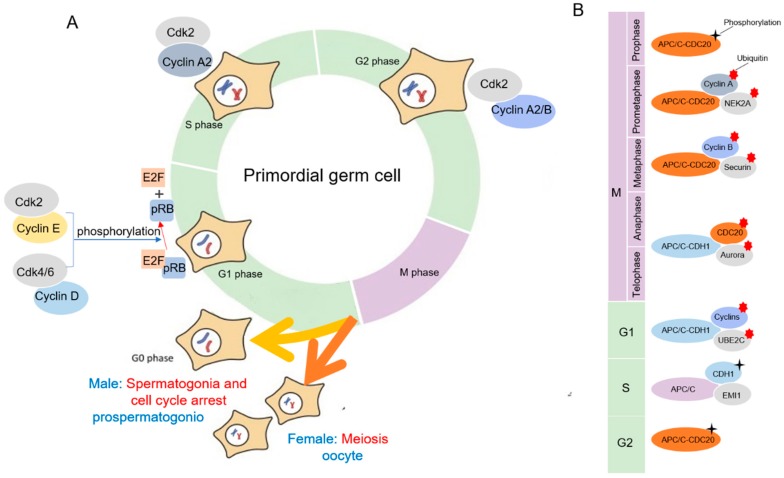
(**A**) Illustration of the main cell cycle genes expressed and likely controlling the cell cycle in proliferating mouse PGCs. (**B**) The role of APC/C in the cell mitosis cycle.

Spermatogenesis is the process through which diploid spermatogenic stem cells grow into haploid sperms in the male testis. There are some indispensable stages, including the mitosis of the spermatogonial stem cell (SSC), meiosis of the spermatocyte, and spermiogenesis. The mitotic division of SSCs is located adjacent to the basement membrane, which produces type A or B spermatogonia (see Figure 1) [23]. Type A spermatogonia replenish the stem cells, and type B spermatogonia develop into spermatocytes [24,25]. A single type A spermatogonia (A_single_ or A_s_) undergoes a self-renewing division to produce two new A_s_ cells. A_s_ spermatogonia, often considered SSCs, must undergo division to produce a pair of spermatogonial cells (A_paired_ or A_pr_). They then differentiate into 4–16 and even 32 spermatogonial cells (A_aligned_ or A_al_) via a series of mitotic cell divisions [26,27]. Due to rapid and incomplete cytokinesis, A_pr_ and A_al_ connect to one another using intercellular bridges (see Figure 2) [27,28]. Studies of the mouse male germline have shown that the cytoplasmic bridges may permit “cytoplasmic sharing” of essential signals for synchronous cell divisions and facilitate the sharing of gene products between post-meiotic haploid spermatids so that genetically distinct spermatids remain phenotypically diploid [29,30]. During the mitosis of type A1 spermatogonia, which differentiate from A_al_ cells, these germ cells located in the basement membrane migrate to the seminiferous tubules [31]. A1 spermatogonia subsequently undergo six mitoses each, forming A2, A3, A4, In, and B spermatogonia. Sertoli cells (SCs) also play a central role in spermatogenesis, providing structural support and nutrition to developing germ cells and producing proteins that influence the mitotic activity of spermatogonia [32,33]. The small kinetochore-associated protein (*SKAP*) is a component of the mitotic spindle, which is essential for faithful chromosome segregation during anaphase [34]. *SKAP^−/−^* mice grow normally without any obvious developmental defects. Therefore, *SKAP* is dispensable for somatic cell divisions in mice. However, *SKAP* affects mitosis in spermatogenesis because *Skap^−/−^* mice have smaller testes and a strong decrease in sperm production before meiosis compared with wildtype mice [35].

## 3. Mitosis of Female Gametogenesis

Oogenesis is the process of female gamete development which takes place in ovaries. It is complex and regulated by a vast number of intra- and extra-ovarian factors [36]. Oogonia, which are generated from PGCs, proliferate by mitosis and form primary oocytes. However, unlike spermatogenesis, oogonia are formed in large numbers from PGCs by mitosis during early fetal development, which then arrest at prophase stage of the first meiotic division around the time of birth [37,38].

## 4. Gene Regulation of Mitosis during Mammalian Gametogenesis

PGCs divide into eggs or spermatids and emerge as clusters of multiple cells that share one cytoplasm in early embryos [39,40]. Then, PGCs propagate rapidly and grow in number but stop propagation during the late pregnancy period in mammals [41]. In this period, female germ cells enter the meiotic prophase instantly, whereas male germ cells subsequently arrest in the G1 phase until puberty. The process of mitosis in gametes is regulated by several genes. Studies have demonstrated that the specific deletion of *Mastl* in mouse PGCs leads to the failure of cells to proceed beyond the metaphase-like stage of mitosis. This mitotic defect results in the activation of the DNA damage response pathway. Thus, the majority of *Mastl*^−/−^ PGCs undergo apoptosis [42]. *Pin1*, a peptidyl-prolyl isomerase, is involved in the regulation of mammalian PGC proliferation. PGCs have a prolonged cell cycle in the absence of *Pin1*, leading to fewer cell divisions and strikingly fewer *Pin1*^−/−^ PGCs by the end of the proliferative phase. Therefore, male and female *Pin1*^−/−^ mice have profound fertility defects [43]. The *PTEN* gene can inhibit cell proliferation via restraint of the PI3K/AKT pathway, as revealed by *Pten*^−/−^ PGCs both in vivo and in vitro. These cells show significantly increased mitosis [44,45,46]. Similarly, Kit (Kit oncogene) and Kitl (Kit ligand or stem cell factor) are also required for the migration and proliferation of PGCs throughout embryogenesis [47,48].

Some DNA repair-associated genes are also involved in the proliferation of PGCs. *Rev7* and *Mcm9* are related to cell cycle regulation and homologous recombination repair by recruiting RAD51 to sites of DNA damage in mammals [49,50,51]. Germ cell depletion is the result of reduced PGC numbers both before and after they arrive in the primitive gonads of *Mcn9* mutant mice [52]. *Rev7*^−/−^ mice display the loss of germ cells by apoptotic cell death during migration and germ cell aplasia in both testes and ovaries after birth [53]. According to these studies, DNA repair-associated genes, such as *Rev*, also regulate gamete mitosis.

In many mammalian species, the *Nanos2* gene encoding RNA-binding proteins was identified as functional in controlling the proliferation of PGCs and maintaining the stemness of undifferentiating SSCs [54]. In male *nanos2/nanos3*-null mice, the size and weight of their testes are reduced and no gamete cells can be detected compared to *nanos2/nanos3*^+/+^ mice [55,56]. These findings suggest that *Nanos* genes are involved in the maintenance of mitosis in gametes by supporting their proliferation and/or suppressing apoptosis. The *Sox4* gene is expressed in gonadal supporting cells, the organizing center of gonad organogenesis. However, Nanos2 in male *Sox4*-null mice is expressed at levels substantially lower than in wildtype testes, indicating that the mitosis of male germ cells is severely impaired in *Sox4*^–/–^ testes [57]. DND1 expressed in mouse germ cells regulates the mitotic arrest of male germ cells through the translational regulation of cell cycle-related genes [58].

### 4.1. Cyclin/CDK Regulation of Mitosis during Mammalian Gametogenesis

Cdks are serine/threonine kinases, and their catalytic activities are modulated by interactions with cyclins and Cdk inhibitors. They have roles in regulating the cell cycle, transcription, and mRNA processing [59]. In mammalian somatic cells, there are at least four different Cdks to regulate the interphase of mitosis: Cdk2, Cdk3, Cdk4, and Cdk6. After interphase, Cdk1 drive cells through mitosis (Figure 2A) [60,61]. To perform kinase activity, CDK must bind to a cyclin as a regulatory protein. Only the cyclin–CDK complex is an active kinase. Using a cell cycle array revealed that PGCs were comparable with somatic cells in the G1 phase, and it included expression of cyclin D3 (ccnd3), the retinoblastoma protein (pRB) family, and CDK inhibitors [62]. According to the mammalian PGC cycle, cyclin E and cyclin D were found to be the predominant cyclins. Cyclin D activates Cdk4/6, and the pRB family is phosphorylated in the early G1 phase. On the contrary, the reason why prospermatogonia undergo mitotic arrest at the G1/S phase checkpoint is that cyclin E1/2 and cyclin B1/2 are downregulated and cyclinB3 is upregulated, which result in the hypophosphorylation of pRB. Then, E2F transcription factors are released, leading to the activation and transcription of E2F-responsive genes for cell cycle progression [63]. During the late G1 phase, Cdk2 is stimulated via binding to cyclin E [64]. A study shows that the presence of lower levels of cyclin E1/E2 and cyclin B1/B2 in the 13.5–14.5 dpc cell cycle arrested prospermatogonia, as compared with mitotic 11.5–12.5 dpc PGCs [62]. Subsequently, prospermatogonia pass the restriction point of the G1/S phase and enter the S phase. Similar to E-type cyclin binding, cyclin A synthesized at the onset of S phase also activates Cdk2 that phosphorylates proteins involved in DNA replication [65]. At the transition phase of G2/M in germinal cells, cyclin A2 binds to Cdk1, which is required for the initiation of prophase. Finally, the cyclin B complex with Cdk1 drives entry into the M phase [66,67].

*Ccna2*, a cyclin A gene, is expressed in the spermatogonia of the adult mouse testis, which may have an S phase function in the mitotic cell cycle of spermatogonial germ cells. During embryogenesis, D-type cyclin expression is primarily restricted to CCND3 in male germ cells [68]. Cyclins D1 and D3 are expressed in spermatogonia at all cell cycle phases of the seminiferous tubule epithelium in the adult testis. However, cyclin D2 is detected at the stage when type A_al_ spermatogonia differentiate into type A_1_ spermatogonia, indicating that cyclin D2 is involved in SSC proliferation [69]. The Cdkn1b-encoded protein binds to and prevents the activation of cyclin E–CDK2 and cyclin D–CDK4 complexes. Cdkn1b^−/−^ SSCs show suppressed proliferation and diminished expression of CDK4 and pRB1, resulting in the poor phosphorylation of pRB1. In cultured SSCs, pRB1 deficiency leads to cell cycle arrest and apoptosis [70]. In mitosis-arrested male germ cells, p27Kip1 and p15INK4b encoded by Cdkn1b and Cdkn2b genes are upregulated, which inhibit CyclinE–cdk2 and CyclinD–cdk4/6, respectively. P27Kip1 and p15INK4b ensure that hypophosphorylated pRB can inhibit the G1/S phase transition and suppress cyclin E expression [63]. pRB1 is required for germ cell entry into G1/0 arrest in the normal gonad. However, in *pRB*^−/−^ mice, upregulation of other cell cycle suppressors, including Cdkn1b and Cdkn2b, can induce delayed germ cell arrest [71].

### 4.2. APC/C Regulation of Mitosis during Mammalian Gametogenesis

The anaphase-promoting complex or cyclosome (APC/C), the E3 ubiquitin ligase, plays an important role in the regulation of the mitotic cell cycle [72]. In the mitotic cell, the spindle assembly checkpoint (SAC) ensures that each daughter cell inherits an identical set of chromosomes. With the activated APC/C, it coordinates the accurate attachment of sister chromatid kinetochores to the spindle [73].

During mitosis in mammalian gametogenesis, the germ cell cycle is controlled by the oscillation in activity of CDKs. Like the somatic cells, this precisely regulated degradation process is accomplished by APC/C catalyzed ubiquitination in germ cells [74]. Cell division cycle 20 (CDC20) and CDC20 homologue 1 (CDH1) are the activating subunits of APC/C (Figure 2B) [74]. The APC/C is inactive from the late G1 phase to early prophase to ensure its main substrates accumulate. The APC/C–CDH1 complex mainly regulates the process of anaphase and the early G1 phase. At the G1/S transition, APC/C–CDH1 is inactivated by a combination of binding to the APC/C inhibitor early mitotic inhibitor 1 (EMI1), degradation of ubiquitin-conjugating enzyme E2C (UBE2C), and CDH1 phosphorylation. APC/C–CDC20 complexes are primarily regulated by the degradation of related substrates in the prometaphase and metaphase phases [75]. In the G2 phase, CDC20 is phosphorylated by CDK1, partially activating the phase using APC/C interaction. In the prometaphase, the activity of the APC/C–CDC20 complex is inhibited during the G2/M phase. The APC/C–CDC20 complex ubiquitinates cell cyclin A and NIMA-related expressed kinase 2A (NEK2A). Cyclin B1–CDK1 complexes and securin keep the cell in the M phase. Thus, in the metaphase, the APC/C–CDC20 complex degrades cyclin B1 and CDK1 to end cell division. In anaphase, CDH1 is dephosphorylated and the APC/C–CDH1 complex is activated. The APC/C–CDH1 complex ubiquitinates CDC20, Aurora A/B, and other kinases to promote the end of mitosis [72].

## 5. Signaling Pathways/Molecules Regulating Mitosis during Mammalian Gametogenesis

SSCs are located in the basal membrane of the convoluted spermatogonia, which can not only undergo self-renewal to maintain a stable SSC number but also generate spermatocytes by directional differentiation [76]. The processes of SSC self-renewal and differentiation are also regulated by some signaling pathways or signaling molecules [77].

**Figure 3:**
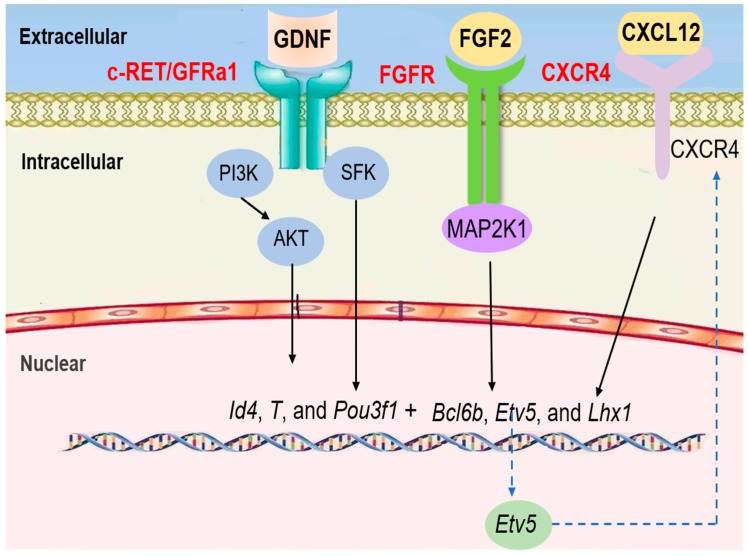
Current understanding of the signaling pathways regulating the mitosis of SSCs in mouse testes.

The steady state of mitosis in SSCs is driven by a complex paracrine and endocrine system within structurally well-organized tissue. Central to this system are SCs that provide nutritional and structural support for the self-renewal and differentiation of germ cells (Figure 3) [78,79]. GDNF, a paracrine factor, is secreted by SCs. It promotes SSC self-renewal and maintenance via mitosis. In mice, GDNF plays a role in promoting spermatogonial self-renewal using RET tyrosine kinase and a ligand-specific co-receptor, GFRα1, which is expressed on undifferentiated type A spermatogonia [80,81,82]. Because GFRαA1 and RET are concomitantly expressed in A_paired_ spermatogonia and some A_aligned_ spermatogonia, GDNF induces and maintains the proliferation of these cells as SSCs begin differentiation [83]. According to single cell RNA-seq data from mice and humans, GFRα1 is mostly expressed in undifferentiated spermatogonia and co-expressed with Pou3f1, Bcl6b, and Etv5 [84,85]. Etv5 is a transcription factor secreted by SCs which can promote the RET synthesis of GDNF receptor and ensure that the GDNF signal pathway is transmitted from the extracellular to the intracellular space [86]. Pou3f1 is a member of the transcriptor OCT combined with the family, which also has an important modulatory function for SSC proliferation [87]. Bcl6b, a member of the poxvirus and zinc finger (POZ) family of transcriptional repressors, is another target of GDNF that was recently identified in SSCs by microarray analysis [88]. When GDNF is applied to a germline stem cell culture, Akt is phosphorylated rapidly, and the addition of a chemical inhibitor of PI3K prevents GS cell self-renewal, indicating that the PI3K/Akt pathway is essential for the self-renewal of spermatogonial stem cells [89]. GDNF binds to GFRα1 on the SSC membranes to form the GDNF–GFRα1 complex, which binds and activates RET. The MAPK, SFK, and PI3K/AKT signaling pathways will be further activated [90]. In SSCs, the phosphorylation of AKT is mainly activated by PI3K, which results in AKT self-activation. The activation of AKT can switch up the expression of transcription factors, such as Etv5, Pou3f1, and Bcl6b, to promote SSC self-renewal [91].

Similarly, the SFK (SRC family kinase) pathway also mediates GDNF functions in SSCs in vitro [92]. Experiments using SSCs treated with GDNF have shown that GDNF increases the number of SSCs and that downstream targets of the GDNF/RET signaling pathway, such as ID4, BCL6, ETV5, and LHX1, are critical for SSC self-renewal [93,94]. GFRα-1 and Ret are expressed in SSCs, and the Src family co-precipitates with Ret after GDNF stimulation. SFK promotes SSC proliferation through Ret activation. Src and Yes play an especially predominant role in the immediate response of primary SSCs to GDNF. Further, Src activates a PI3K/Akt signaling pathway and switches up the expression of transcription factors, such as Etv5, Pou3f1, and Bcl6b [95].

FGF is also a bona fide mitosis factor for the self-renewal of spermatogonial stem cells. In vitro studies have shown that FGF2 promotes mitogenic effects, and the differentiation of SSCs can be blocked by the addition of FGF2 to culture medium, although it’s in vivo effects are unclear [96,97]. FGF2 relies on MAP2K1 activation to drive SSC self-renewal via upregulation of Etv5, Bcl6b, and Lhx1 genes, according to a mouse germline stem cell culture system that allows the in vitro expansion of SSCs [98]. Similar to FGF2, FGF5 promotes the proliferation of cultured GFRα1^+^ spermatogonia and mouse SSC line C18-4 in a time- and dose-dependent manner via ERK and AKT activation [99,100]. FGF5 also upregulates genes associated with self-renewal, such as Etv5, Id4, and Shisa6 [101,102,103]. CXCL12, a chemokine protein, specifically binds to the CXCR4 receptor, which is expressed in Sertoli cells to regulate SSC self-renewal and maintenance. The signaling response to CXCL12–CXCR4 activation is involved in the prevention of the transition to a progenitor state, the regulation of SSC proliferation, and the guidance of SSC homing to cognate niches [104].

Taken together, GDNF and FGF2 signaling work in concert to activate PI3K/AKT, SFK, and MAP2K1, which are critical regulators to switch up the expression of transcription factors. Furthermore, the transcription factor Etv5 regulates CXCR4 expression in SSCs, thereby controlling the signaling response of CXCL12 to influence self-renewal, proliferation, and homing [105].

## 6. mRNA Regulation in Mitosis during Mammalian Gametogenesis

During the mitosis of mammalian gametes, microRNAs (miRNAs), small non-coding RNAs 18–23 nt in length, are viewed as active regulators in the post-transcriptional regulatory processes of germ cells [106,107]. Dicer, as an RNase III endonuclease, plays a critical role in the biogenesis of miRNAs [108]. In the Dicer-deleted testis, PGCs and spermatogonia exhibit poor proliferation [109]. Here, we discuss some specific roles of a few miRNA molecules in mitosis during gametogenesis (Table 1). miR-17-2 is highly expressed at the stage of PGC development, which regulates the mitosis of PGCs. Moreover, miRNA-17-5p, -18, -19a, and -19b, which are only expressed in females, regulate PGC exit from mitotic proliferation [110]. MiR-19a and -19b may regulate *PTEN* dosage, which negatively controls PGC proliferation [111]. In a recent study, miRNA-31-5p mimics decreased the level of cyclin A2 rather than cyclin D1 or cyclin E1, which regulates the proliferation and DNA synthesis of human SSCs via the PAK1-JAZF1-cyclin A2 pathway [112]. The miR-290-295 cluster is only present in placental mammals. It consists of seven miRNA precursors: miR-290, miR-291a, miR-292, miR-291b, miR-293, miR-294, and miR-295. The miR-290-295 cluster affects the cell cycle of PGCs at multiple points. Under certain conditions, it might assist G1/S progression and regulate the G2–M transition of PGCs and ES cells [110,113]. MiR-302 family members were specifically expressed in PGCs, and the validated target gene is the cyclin-dependent kinase inhibitor 1A (*Cdkn1a*). MiR-302 downregulated *Cdkn1a* to ensure that PGCs enter the G1/S transition of mitosis [114]. MiR-202 family members, including miR-202-3p and miR-202-5p, are highly expressed in mouse spermatogonial stem cells (SSCs) and are oppositely regulated by GDNF, a key factor for SSC self-renewal. By using CRISPR/Cas9-mediated knockout of miR-202 in cultured SSCs, a study found that miR-202^−/−^ SSCs initiate premature differentiation, accompanied by reduced stem cell activity and increased mitosis [115]. Dmrt1 determines whether male germ cells undergo mitosis and spermatogonial differentiation or meiosis by controlling cyclical gene expression in Sertoli cells [116]. MiR-224 targets the DMRT1 3′-UTR, and the overexpression of miR-224 influences SSC differentiation by suppressing DMRT1 expression [117].

## 7. Concluding Remarks

We have systematically summarized the regulation of genes, cyclins/CDK, signaling molecules, and miRNAs in mitosis during gametogenesis. PGCs undergo rapid mitosis during migration to the reproductive ridge, and spermatogonial stem cells undergo mitosis during self-renewal and differentiation [118]. In recent years, with the deepening of research and technological progress, we are gradually expanding our knowledge of mitosis during gametogenesis, although specific mitosis mechanisms of gametes are still unclear. For example, it remains to be revealed how different intracellular pathways activated by external signals are integrated and functionally associated with cell cycle cyclins, CDK controllers, and miRNAs in mitosis of gametes [64]. In addition, it is unknown how male, but not female, mouse PGCs enter mitotic arrest at 13.5–14.5 dpc. To further explore these aspects, in vitro reconstitution of mouse germ cell development from mPSCs may provide a robust foundation to understand the mitosis mechanism of germ cells, including signaling pathways, transcriptional networks, and epigenetic regulation. In the near future, omics analysis and structural biology may also play indispensable roles in resolving basic issues in gamete mitosis. Many molecules involved in the regulation of mitosis during gametogenesis have been identified; however, further investigation will be essential to elucidate the signaling pathways assigned for maintenance of undifferentiated state, self-renewal, and differentiation [82]. In the future, the functions of putative mitosis during gametogenesis molecules and signaling pathways need to be verified via a functional transplantation assay.

## Figures and Tables

**Table 1:** miRNA molecules implicated in germ cell mitosis.

Name	Expression	Proposed Function	Targets Involved in Mammalian Gametogenesis	Reference
miR-17-92 cluster	PGCs, ES cells	Regulator of differentiation, proliferation and apoptosis	STAT3, E2F1, PTEN	[110,118,119]
miRNA-31-5p	SSCs	Regulator of SSCs proliferation	JAZF1 and Cyclin A2	[112]
miR-290-295 cluster	PGCs	G1 to S phase cell cycle control	WEE1, FBXL5	[110,113]
miR-202	SSCs	Regulator of cell cycle and apoptosis of mitosis	Rbfox, Cpeb1	[115]
miR-224	SSCs	Control SSCs self-renewal and cyclical gene expression	DMRT1	[117]
MiR-302-67 cluster	PGCs	targeting inhibitors of the G1/S transition	Cdkn1a	[114,120]
miR-125a	Later male PGCs	Control of differentiation	LIN28	[110]
miR-200c	Early PGCs	Control of apoptosis	ZEB1, TRKB	[110,121]
miR-21	SSCs	SSCs self-renewal, anti-apoptosis	ZEB1, TRKB	[122]
miR-221	PGCs	Regulate mitotic arrest in male germ cells	DND1	[123]
miR-34c	PGCs	Cell cycle regulator	CCND3, CCNG1, CCNB1 NOTCH2	[124,125]

## Abbreviations

APC/C	anaphase-promoting complex or cyclosome
Akt	thymoma viral proto-oncogene 1
ATF	activating transcription factor
Bcl6b	B-cell CLL/lymphoma 6, member B
Ccnd	cyclin D
CDC20	cell division cycle 20
CDH1	CDC20 homologue 1
Cdk	cyclin-dependent kinase
CXCL12	chemokine (C-X-C motif) ligand 12
CXCR4	C-X-C chemokine receptor type 4
EMI1	early mitotic inhibitor 1
Etv5	ets variant gene 5
Fgf2	fibroblast growth factor 2
Gdnf	glial cell line-derived neurotrophic factor
Gfrα-1	glial cell line-derived neurotrophic factor family receptor alpha 1
Kitl	KIT ligand or stem cell factor
NEK2A	NIMA-related expressed kinase 2A
PI3K	phosphatidylinositol 3-kinase
POZ	poxvirus and zinc finger
pRB	retinoblastoma protein
SCs	Sertoli cells
SFK	src family kinase
SKAP	Small kinetochore-associated protein
SSC	spermatogonial stem cell
Taf4B	TATA box-binding protein (TBP)-associated factor, subunit 4B
UBE2C	ubiquitin-conjugating enzyme E2C
